# Stability of Neural Firing in the Trigeminal Nuclei under Mechanical Whisker Stimulation

**DOI:** 10.1155/2010/340541

**Published:** 2010-01-06

**Authors:** Valeri A. Makarov, Alexey N. Pavlov, Anatoly N. Tupitsyn, Fivos Panetsos, Angel Moreno

**Affiliations:** ^1^Departamento de Matemática Aplicada, Universidad Complutense de Madrid, Avenida Complutense s/n, 28040 Madrid, Spain; ^2^Radiophysics and Nonlinear Dynamics Chair, Physics Department, Saratov State University, Astrakhanskaya Str. 83, Saratov 410026, Russia; ^3^Neurocomputing and Neurorobotics Group, Universidad Complutense de Madrid, Avenida Arcos de Jalón s/n, 28037 Madrid, Spain

## Abstract

Sensory information handling is an essentially nonstationary process even under a periodic stimulation. We show how the time evolution of ridges in the wavelet spectrum of spike trains can be used for quantification of the dynamical stability of the neuronal responses to a stimulus. We employ this method to study neuronal responses in trigeminal nuclei of the rat provoked by tactile whisker stimulation. Neurons from principalis (Pr5) and interpolaris (Sp5i) show the maximal stability at the intermediate (50 ms) stimulus duration, whereas Sp5o cells “prefer” shorter (10 ms) stimulation. We also show that neurons in all three nuclei can perform as stimulus frequency filters. The response stability of about 33% of cells exhibits low-pass frequency dynamics. About 57% of cells have band-pass dynamics with the optimal frequency at 5 Hz for Pr5 and Sp5i, and 4 Hz for Sp5o, and the remaining 10% show no prominent dependence on the stimulus frequency. This suggests that the neural coding scheme in trigeminal nuclei is not fixed, but instead it adapts to the stimulus characteristics.

## 1. Introduction

The rodent vibrissae system is one of the most used experimental models for the study of the sensory information handling. Rats actively use their whiskers to detect and localize objects and also to discriminate surface textures. By sweeping whiskers at rates of 5–20 Hz, they can localize objects within a few whisking cycles or even with a single vibrissa [1]. Thus relatively short temporal, but not spatial mechanical information, dominates in the object detection. Similarly, the texture mechanical coding, attributed to the whisker resonance (i.e., the vibration amplitude across the whisker array codifies the texture [2]), occurs in awake rats and shapes natural whisker vibration. However, it seems that textures are not encoded by the differential resonance. Instead, slip-stick events contribute to a kinetic signature for texture in the whisker system, which suggests the presence of a temporal structure in neural spike trains under these experimental conditions [3].

Thus the efficacy of the sensory information transmission and processing in the time domain resides in the possibility of multiple cells to generate coherent responses to a stimulus, which constitutes the neural code. Though there was a long discussion about what type of the neural code is employed by each individual neuron or neuron group, the growing experimental evidence shows that a same neuron may use different coding schemes (see reviews in [4, 5]). The temporal correlation of multiple cell discharges has been also found important for the information transmission to the cortex and its modulation by the corticofugal feedback (see, e.g., [6–8] and references therein).

**Figure 1:**
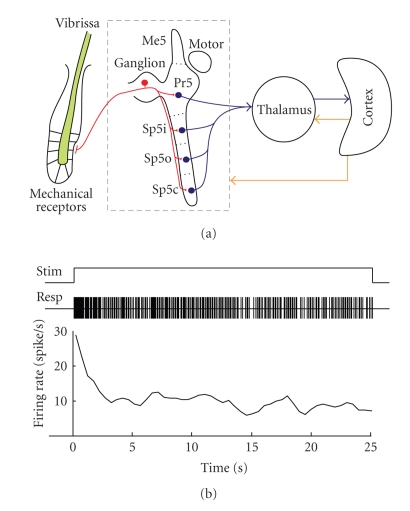
(a) Sketch of the main steps in the tactile information processing. (b) Firing rate dynamics of a neuron from Pr5 nucleus under a periodic (10 Hz) stimulation of a vibrissa in the neuron receptive field.

The large mystacial vibrissae of the rat or “whiskers” are arranged in an array of five rows and up to seven arcs. Somatosensory information from the whiskers arrives to the trigeminal complex organized into one motor and three sensory nuclei including the principal nucleus or principalis (Pr5), the spinal nucleus (Sp5), and the mesencephalic nucleus (Figure 1(a)). In turn Sp5 consists of three subnuclei called oralis (Sp5o), interpolaris (Sp5i), and caudalis (Sp5c). Information from Pr5 and Sp5 goes to the contralateral thalamus (VPm) and then to the primary somatosensory (SI) cortex. There is also a feedback monosynaptic projection with an extremely precise somatotopy from SI to the trigeminal nuclei. Over whole pathway primary afferents and neurons form spatial structures (barrelettes, berreloids, and barrels, in the trigeminal complex, VPm and SI, resp.), which replicate the patterned arrangement of the whisker follicles on the snout (for details see, e.g., [9–12]).

Even under a periodic, completely repeatable stimulus, the neuronal response in the trigeminal nuclei is far from being constant. Figure 1(b) shows an example of firing dynamics of a neuron from Pr5 under a periodic stimulation of a vibrissa in its receptive field (RF). During first few seconds, the neuron exhibits the maximal firing rate (about 27 spikes/s), then the rate quickly falls down to about 10 spikes/s, and further fluctuates for more than 20 s. Thus the neuron behavior is essentially nonstationary.

Traditional analysis of neural spike trains has often been performed assuming that segments of the experimental time series are approximately stationary and that such segments can be studied by means of statistical techniques such as, for example, correlation measures or Fourier analysis (see, e.g., [13–16]). This approach is obviously useful if the nonstationarity has a time scale longer than the rhythms of interest. However, this situation is not always true. Instantaneous frequencies of various rhythmic components can exhibit complex and irregular fluctuations, that is, the nonstationarity may be associated with higher frequencies as well. Our previous results [6] showed that the Fourier analysis is hardly applicable in such conditions. An alternative is the use of the wavelet technique, which can be successfully applied for analysis of the temporal structure of the neuronal spiking in a wide range of time scales [6, 17].

In this paper we study the dynamical stability of the spiking patterns in the trigeminal nuclei evoked by a periodic mechanical whisker stimulation. We show that the analysis of spectral ridges in the wavelet space of spike trains enables classification of neurons depending on the degree of repeatability of their response patterns. We also show that the stability of response patterns of a single neuron varies depending on the stimulus properties. This result contrasts with the robust temporal coding in the trigeminal ganglion [18] and suggests that the coding scheme in Pr5 and Sp5 nuclei is not fixed, but instead this relay is adaptable and performs integration of the sensory information.

## 2. Processing of Neural Spike Trains

For information processing, it is reasonable to assume that neurons produce and exchange stereotypical events; thus, only timings of the spike occurrences carry a message. Consequently before applying any analysis, we first identify and normalize spikes in experimental data.

### 2.1. Preliminary Analysis of Extracellular Potential

**Figure 2:**
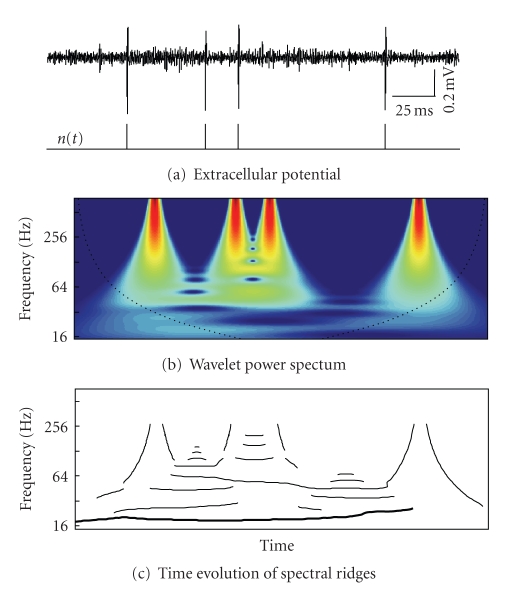
Wavelet analysis of a spike train. (a) Conversion of extracellular recording into a spike train, *n*(*t*). (b) Energy density *E*(*f*, *t*) of the spike train (color from blue to red corresponds to the spectrum magnitude). Dashed black curve defines the cone of influence where the edge effects canot be neglected. (c) Time evolution of “spectral ridges” *F*_*k*_(*t*). Thick curve corresponds to the main (most prominent and stable) ridge, whose central frequency varies in time around 20 Hz.

Extracellular recordings usually represent a multiunitary neuronal activity, where several cells nearby the electrode tip produce short lasting electrical pulses or spikes different in shape and amplitude. Thus before analyzing the firing dynamics of a single neuron, its spikes should be isolated from the activity of the other cells. Figure 2(a) illustrates a typical example of a high-pass filtered (*f*_cut_ = 300 Hz) experimental recording made in Pr5 nucleus. Four spikes coming from a single cell are seen by the naked eye. However, in more complex situations, advanced spike sorting techniques including those based on the wavelet transform can be used (see, e.g., [19, 20] and references therein). Here we applied the wavelet shape-accounting classifier (WSAC) [20] to separate and sort high-amplitude spikes.

Once spikes of a single cell have been identified, we represent them as a series of *δ*-functions

(1) $$n\left(t\right)=\sum \delta \left(t-t_{i}\right),$$

where *t*_*i*_ are the time instances of spike occurrences (Figure 2(a)).

### 2.2. Wavelet Power Spectrum of Spike Train

Let us shortly recall the theoretical background of the wavelet analysis of a spike train (point process) *n*(*t*) (details can be found in [6, 17]). The continuous wavelet transform is given by

(2) $$W\left(p,t\right)=\frac{1}{\sqrt{p}}\int n\left(\tau \right)\psi \left(\frac{\tau -t}{p}\right)\text{d}\tau ,$$

where, for the spectral analysis, *ψ*(*y*) = *e* ^*j*2*π y*−*y*^2^/2*k*_0_^2^^ is the Morlet function, *n*(*t*) is the spike train (1), and *p* is the time scale. The wavelet parameter *k*_0_ can be adjusted according to the problem (see, e.g., [6]). Here we used *k*_0_ = 1 to balance the time-frequency resolution. The wavelet time scale *p* is linked to the frequency content of the spike train by *f* ≈ *k*_0_/*p* (if *k*_0_ ≥ 1).

The time evolution of the energy density of the spike train *n*(*t*) can be estimated as

(3) $$E\left(p,t\right)=\frac{1}{\sqrt{\pi }rk_{0}p}{\left|W\left(p,t\right)\right|}^{2},$$

where *r* is the mean firing rate (normalization of the energy spectrum per spike simplifies comparison of neurons with different firing rates). For biophysical convenience instead of (3), we shall use its frequency counterpart *E*(*f*, *t*) by substituting *p* = 1/*f* (*k*_0_ = 1).

*E*(*f*, *t*) represents a surface in 3D space, whose sections at fixed time moments correspond to the local energy spectra. Figure 2(b) shows 2D plot of the energy density of the spike train shown in Figure 2(a). Each spike produces a wide frequency spectrum. The existence of rhythms in the spike train leads to appearance of “*ridges*” in the 3D energy surface, associated with the rhythmic contributions. These ridges, oriented along the time axis, identify spectral content of the spike train at a given time moment.

Thus the dynamics of rhythmic components hidden in a spike train is reflected in the time evolution of spectral ridges. To construct spectral ridges, we identify local spectral maximums at each vertical section t* of the energy spectrum *E*(*f*, t*) (Figure 2(b)), thus obtaining a set of 2D functions of time *F*_*k*_(*t*), where the subindex corresponds to the ridge number (Figure 2(c)).

### 2.3. Stability Measure of Neuronal Response

Spectral ridges can appear and disappear in time and also oscillate (Figure 2(c)), which indicates the presence of a given rhythm in the spiking dynamics of a neuron and its interaction with other rhythms (e.g., due to a high frequency jitter of spike timings). Under a periodic stimulation, for a stereotypic neuronal response, that is, when the neuron generates the same pattern per each stimulus event, its instantaneous frequency associated with the stimulus rhythm remains constant. Thus we shall observe a “perfect” (continuous and straight) spectral ridge at the stimulus frequency.

Deviation from the stereotypic response (e.g., due to missing spikes or changes in interspike intervals) causes “floating” of the instantaneous (up to the time resolution) frequency in time and even disappearance of the ridge, similarly as it happens in Figure 2(c). Moreover, the stronger the fluctuation of the instantaneous frequency, the more significant differences in neuronal responses take place. Thus following the time evolution of the instantaneous frequency of spectral peaks enables studying the stability and stationarity of neuronal responses to a tonic stimulus.

To quantify the neuronal response stability, we introduce the following measure:

(4) $$S=\frac{1}{\sigma_{0}},$$

where *σ*_0_ is the standard deviation of the time evolution of the main ridge *F*_0_(*t*) found in a vicinity of the stimulus frequency.

To evaluate *S* for a spike train, first we calculated its energy density (3). Then for a fixed time t* (changed with 5 ms time bin), we searched for the energy maximum in the frequency range *f*_stim_ ± 5%. The obtained frequency was assigned to *F*_0_(t*). Finally, the standard deviation of *F*_0_ yields *S*.

## 3. Dynamical Stability of Neuronal Response

Let us exemplify the proposed methodology for studying the stability of neuronal responses to an external stimulus by using simulated recordings.

We consider three neurons embedded into a network and receiving the same periodic (1 Hz) sequence of 50 stimuli. Depending on the current network state and its dynamics, the neuronal responses may have different variability, that is, the firing patterns provoked by each stimulus event may have different degree of repeatability.

We simulated neuronal responses under three different conditions.

N1: Constant (strong) variability. The neuron responds to each stimulus by generating 3–5 phasic spikes (3.9 ± 1.2 std) with fluctuating spike timings (8 ms std).
N2: Changing (small) variability. The neuron generates a spike train similar to N1, but the firing rate decays linearly (from 5 spikes/stimulus at the beginning to about 2.5 at the end).
N3: Increasing (intermediate) variability. The spike train is similar to N2, but the fluctuation of spike timings increases from 0 at the beginning to about 40 ms std at the end.

The first neuron has a stationary distribution of the response patterns, whereas the second and the third show the stimulus adaptability similar to the experimentally observed (Figure 1(b)). Their firing rates decay in time. The difference between the neurons is in the variability of the spike timings. The neuron N2 has constant fluctuations, whereas the fluctuation magnitude of N3 increases with time.

**Figure 3:**
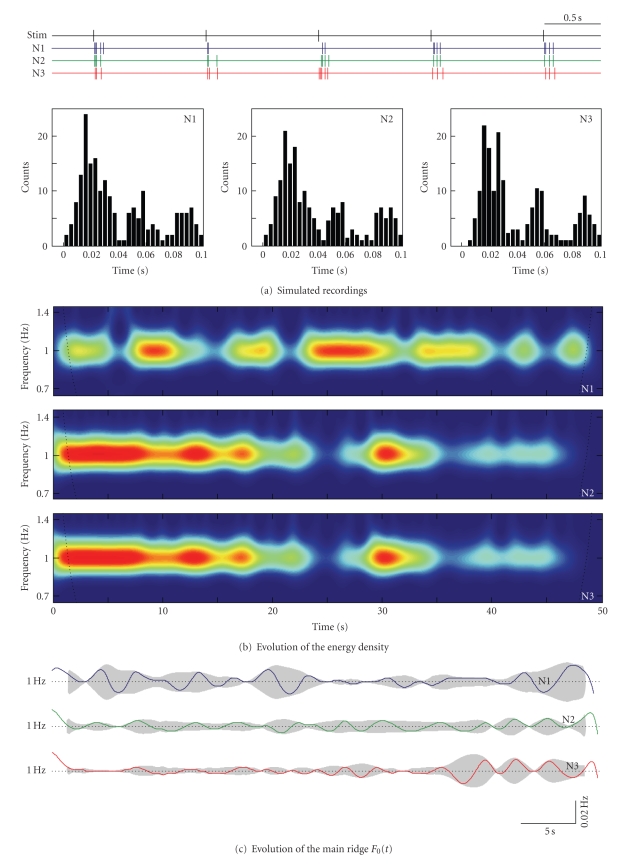

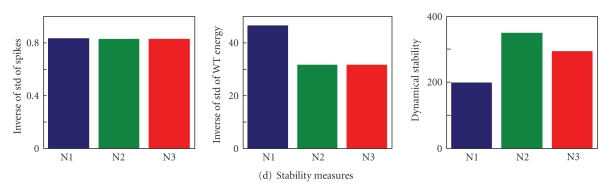
Quantification of the dynamical stability of the stimulus response patterns for three neurons. (a) Stimulus and spike trains (only 5 s epoch shown) of three neurons. The three neurons have fairly the same PSTHs, but their firing dynamics is significantly different (see the main text). (b) Wavelet energy spectra of the spike trains in the stimulus frequency band (color from blue to red corresponds to the spectrum magnitude). (c) Time evolution of the main spectral ridges for the three spike trains. Shadow areas correspond to the envelops of *F*_0_(*t*) (obtained by the Hilbert transform). (d) Different response stability measures: (left) Inverse of the standard deviation of the number of spikes; (middle) Inverse of the standard deviation of the magnitude of the energy density at 1 Hz; and (right) The dynamical stability factor *S*. The latter characteristics reveals distinctions in the stimulus responses of the neurons.

Figure 3(a) shows 5 s epoch of the stimulus and spike trains of the three neurons. Applying the traditional Peri-Stimulus-Time interval analysis, we obtain fairly the same PST histograms. All histograms have three peaks at latencies 20, 50, and 90 ms, corresponding to the neuronal phasic response to the stimulus, and are hardly distinguishable. Thus PSTH fails to quantify the differences in the behaviors exhibited by the neurons, as expected. No much additional information is provided by the raster plot either (not shown).

The wavelet energy spectrum of the first spike train differs significantly from the spectra of N2 and N3, which are very similar (Figure 3(b)). Fluctuation of the spectral magnitude of the 1 Hz rhythm reflects changes in the strength of the neuronal response at that frequency. Loosely speaking, it is proportional to the number of spikes generated per stimulus. The spectral magnitude of the train N1 fluctuates around the mean value, which agrees with the stationary nature of the firing patterns of this neuron. The energy magnitude of N2 and N3 decays in time, again as it was expected according to the decaying firing rate of these neurons.

Figure 3(c) shows the time evolution of the main spectral ridges *F*_0_(*t*) (corresponding to *f*_stim_ = 1 Hz) for the three neurons. It provides information on the phase (temporal) relationships between spikes in the firing patterns and has apparent distinctions in the three cases. The instant ridge frequency of N1 has strong stationary deviations from 1 Hz due to the constant variability of spike timings and “missing” spikes. The ridge of N2 has lower deviations, especially at the first half of the recording, where the neuronal response was more consistent (in the number of generated spikes). N3 shows the smallest ridge variability (close to zero by construction) at the beginning of the stimulation, which progressively grows to the end. The difference with N2 is explained by the growing in time variability of the spike timings of N3.

It is noteworthy that the time evolution of the spectral magnitude (Figure 3(b)) and the ridge dynamics (Figure 3(c)) provide complementary information on the firing patterns. Indeed, a strong neuronal response with similar number of spikes produces a quite stable, high magnitude spectral ridge. If the variability of spike timings is much lower than the inverse of the ridge frequency (interstimulus intervals), then it makes little contribution to the ridge height. However, this high-frequency dynamics will affect the ridge instant frequency and, consequently, will be visible in the *F*_0_(*t*) plot.

Let us now check different measures of the response stability of the neurons N1–N3 that can be derived from the spike trains and their wavelet analysis. First we calculated the standard deviations of the number of spikes elicited by each stimulus. Similar characteristics have been used in [21] for quantification of the frequency-dependent response in VPm and SI neurons. Figure 3(d) (left inset) shows that the inverse of the standard deviation (i.e., 1/std number of spikes) is the same for all neurons, and hence this measure cannot distinguish dynamical differences in their responses.

Figure 3(d) (middle inset) shows the inverse of the standard deviation of the magnitude of the energy density (corresponding to Figure 3(b)) at the stimulus frequency. This measure differentiates the responses of N1 from those of N2 and N3. The lower value for N2 and N3 is mostly due to the trend of the energy magnitude in these cases. Detrending the energy density functions raises the measure to 74 for N2 and N3 and does not affect its value for N1. Thus the energy magnitude-based measure can be a good predictor of the rate neural code; however, it cannot catch the variability in the spike timings.

Finally, Figure 3(d) (right inset) shows the dynamical stability measure (4) evaluated for the three neurons. This measure correctly quantifies the differences between the stability of the firing patterns in all three neurons.

## 4. Stimulus Responses of Trigeminal Neurons

### 4.1. Experimental Methods

Experiments were performed on anesthetized (urethane, 1.5 g/kg) Wistar rats of either sex weighing 200–250 g. The experimental procedure was similar to the described elsewhere [22]. Animals were placed in a stereotaxic device that allowed easy access to the vibrissae. Recordings were obtained using tungsten microelectrodes (0.9–2.0 MΩ) directed vertically into Pr5, Sp5i, and Sp5o nuclei. Once an electrode had been settled, the vibrissae were manually stimulated by means of a thin brush to determine their RFs. The vibrissa maximally activating a neuron nearby the electrode was further used for mechanical stimulation.

Free whisker movements were generated by air puffs directed to one vibrissa only and signals were not recorded when other vibrissae exhibited any vibration. Air pulses were generated by a pneumatic pressure pump (Picospritzer III, Parker Inst. TX) and delivered via a silicon tube, 0.5 mm in diameter, positioned at 10–12 mm perpendicularly to the vibrissa.

- Stimulus protocol S1: three separate sequences of 50 air puffs lasting 10, 50, or 100 ms each with 1 s interpuffs intervals were delivered at the neuron's RF.
- Stimulus protocol S2: air puffs of the fixed duration (10 ms), but with different stimulation frequency, ranging from 1 to 30 Hz, were delivered at the neuron's RF. In the course of individual experiments, the frequency was randomly changed. The whole duration of stimulation with a given frequency was 50 s.

The extracellular potential was amplified, sampled at 20 kHz, passed through the band-pass filter (0.3–3.0 kHz), and then analyzed using the special software Spike 2 and custom packages written in Matlab. For the wavelet analysis, we selected only those neurons whose extracellularly recorded spikes were well isolated from the activity of the other neurons.

### 4.2. Effect of Stimulus Duration (Protocol S1)

**Figure 4:**
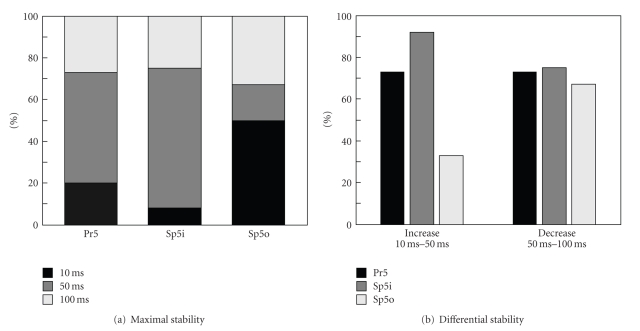
Population analysis of the dynamical stability of the neuronal response patterns under variation of the air puff duration (stimulus protocol S1). (a) Percentage of cells showing maximal stability for 10, 50, or 100 ms stimuli. Neurons from Pr5 and Sp5i “prefer” 50 ms, whereas Sp5o shows better stability for shorter (10 ms) stimuli. (b) Percentage of the neurons showing an increase (left) or decrease (right) of the response stability under increasing the stimulus duration.

We calculated the stability parameter *S* for all selected neurons and the three stimulus durations. Further, we determined the stimulus duration (10, 50, or 100 ms) leading to the maximally stable response pattern for each neuron. To describe quantitative changes of the stability parameter when the stimulus duration increases (10 → 50 → 100 ms), we counted the neurons satisfying the conditions *S*_50_ > *S*_10_ and *S*_50_ < *S*_100_. Figure 4 summarizes results.

In the case of Pr5 neurons, the stability parameter *S* is likely to be maximal for the middle stimulus duration (50 ms, Figure 4(a)). The most stable response is observed for 53% of all cells at 50 ms stimulus. The remaining 27% and 20% of cells stably respond to 100 ms and 10 ms stimuli, respectively.

Quite similar behavior takes place for Sp5i neurons. Here even more cells (67%) “prefer” stimuli of the intermediate duration. This is achieved mostly by decreasing to 8% the cell portion showing better response to the shortest 10 ms stimuli.

Sp5o neurons typically behave differently. The maximally stable response pattern at 50 ms stimulation was observed for only 17% of the cells. Meanwhile, half of the neurons showed better stability at the shortest (10 ms) stimulation. The portion of the cells with better response to the 100 ms stimuli was about 33%, a little bit higher than for Pr5 and Sp5i neurons.

Figure 4(b) shows differential stability characteristics. For 73% of Pr5 neurons, responses to 50 ms stimulation are more stable than those to air puffs of 10 ms duration. In the case of Sp5i neurons, the value of *S* increases at the transition 10 → 50 ms for about 92% of cells. Thus, Pr5 and Sp5i neurons are characterized by rather similar type of reaction to variation of the stimulus duration. However, a different behavior is observed for Sp5o-neurons. Only for 33% of cells *S* increased with the stimulus duration (from 10 to 50 ms). If the stimulus duration increases further (50 → 100 ms), about 70% of neurons from all nuclei decrease their response stability.

Thus the protocol S1 allowed us to conclude that (i) the stability of response patterns depends on the stimulus duration, that is, neurons process differently stimuli of different duration and (ii) there exist significant changes in the types of responses for Pr5, Sp5i and Sp5o neurons, that is, the most reliable responses are achieved in Pr5 and Sp5i for 50 ms stimulus and in Sp5o for 10 ms.

### 4.3. Effect of Stimulation Frequency (Protocol S2)

Let us now study how the response stability depends on the stimulus frequency.

**Figure 5:**
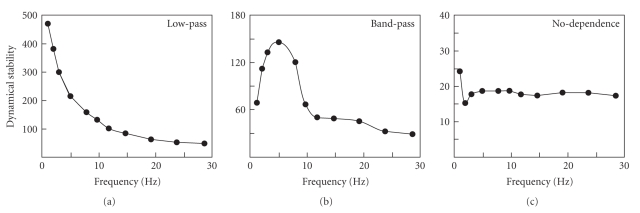
Three main types of behavior of the dynamical stability of the neuronal responses to a tonic stimulus *S*(*f*_stim_).

We found that all trigeminal neurons can be subdivided into three groups by the type of their responses to the frequency content of the stimulus. Figure 5 shows the stability measure as a function of the stimulus frequency *S*(*f*_stim_) for three representative cells. By analogy to the filter terminology, we call the three basic types of the neuronal responses to the frequency content low-pass, band-pass, and no-dependence.

**Figure 6:**
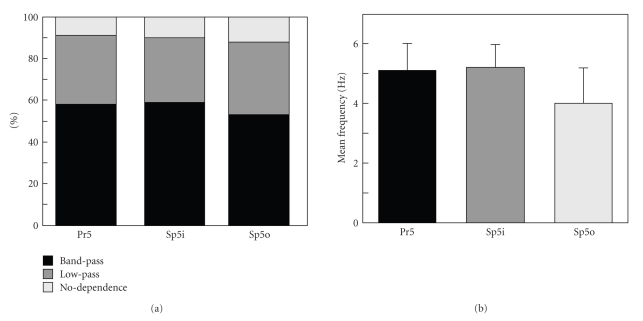
Population analysis of the dynamical stability of the neuronal responses under variation of the stimulus frequency (stimulus protocol S2). (a) Percentage of cells showing different “filtering” characteristics in Pr5, Sp5i, and Sp5o nuclei. (b) Mean central frequencies of the “band-pass” neurons.

In all nuclei the most frequent cell behavior is the band-pass. It occurs in 58%, 59%, and 53% of neurons in Pr5, Sp5i, and Sp5o, respectively (Figure 6(a)). The “low-pass” reaction is observed for 33%, 31%, and 35% of neurons from Pr5, Sp5i, and Sp5o, respectively. Finally, 9%, 10%, and 12% of cells in the corresponding nuclei are characterized by no-dependence reaction. Thus, there are small population distinctions in the frequency filtering properties between Pr5, Sp5i, and Sp5o nuclei.

For the band-pass type responses, we determined the mean central frequency (mean  ± s.e.): 5.1 ± 0.9 Hz (Pr5), 5.2 ± 0.8 Hz (Sp5i), and 4.0 ± 1.3 Hz (Sp5o) (Figure 6(b)). Thus, neurons in Pr5 and Sp5i nuclei have the same central frequency, whereas cells in Sp5o typically show a smaller value of the stabilization frequency.

## 5. Discussion

Ideally, stimulus perception should be invariant to the details of the whisking motion and neuronal responses underlying the object discrimination should carry information specific to the object. This requires flexibility and adaptability in the processing of the whisker vibrations. Recently, it has been shown in vitro [23] that barrel cortex neurons indeed adapt their input-output function, such that the gain rescales depending on the range of the current stimulus distribution. In this paper we have shown that in vivo accommodation of the firing patterns to the stimulus characteristics can be quantified by the novel stability measure *S*, which we used to study neuronal responses in the trigeminal nuclei evoked by tactile whisker stimulation.

From the viewpoint of the analysis of neural spike trains, the attractiveness of a particular technique depends on its generality, for example, on the lack of restrictions on the train stationarity. We have shown that the analysis of the time evolution of frequency ridges in the wavelet space allows identification of the variable frequency content in a neural spike train under essentially nonstationary conditions of the sensory information processing. The method allows an integral quantification of the variability in the number of phasic spikes and in the spike timings, that is, it takes into account both changes at the stimulus time scale and at the significantly shorter time scales. The validity of the method has been cross checked employing simulated spike trains resembling properties of real recordings (Figure 3).

A fundamental issue in neural coding is the role of variation of spike timings in the information processing. Indirectly this can be tested by an artificial jittering of the spike timings and its influence on the derived measures (see, e.g., [18, 24]). The use of the stability measure *S* permits to answer directly the question: how stable or repeatable are the firing patterns produced by a neuron to each stimulation? If the stability measure *S* is high, then the spike patterns are highly repeatable during the whole recordings and, consequently, such a neuron is likely using a temporal code. Conversely, low stability points to a high variability in the spike patterns and suggests the rate code or the presence of a complex dynamics, for example, due to involving local and global feedbacks and fast adaptation.

Previous results [18] showed that the trigeminal ganglion neurons produce a robust and reliable spike trains to whisker deflections, that is, they use temporal code. It has been shown that complex whisker deflections can be reliably predicted by a linear kernel applied to the spike trains recorded from an individual neuron. Here, using the dynamical stability measure, we have shown that neurons in Pr5, Sp5i, and Sp5o nuclei can vary their response stability according to the stimulus characteristics for example, the stimulus duration (Figure 4). Thus the trigeminal neurons adapt their coding scheme depending on the stimulus characteristics, and a continuous oscillation between the two extremes: the temporal and rate codes take place. This conclusion is indirectly supported by the presence of an extensive network locally connecting neurons in the trigeminal nuclei and the global corticofugal projections, that is, the global network dynamics can modify the stimulus evoked patterns of each individual neuron.

Using the stimulus protocol S1 (fixed frequency, variable duration), we found that the response dynamics in Pr5 and Sp5i nuclei is relatively similar, the maximal stability is reached at intermediate stimulus duration (50 ms). It contrasts with Sp5o neurons exhibiting stable responses at shorter (10 ms) stimulation. This indicates significant differences in the adaptation to the same whisker deflections in different nuclei. Partially it can be explained by the differences in the afferent connections and spatial extensions of RFs between the nuclei.

Using the stimulation protocol S2 (variable frequency, fixed duration), we have shown that the stimulus frequency *f*_stim_ influences the neuronal response stability in a nontrivial way. Indeed, one could argue that at low enough frequency (e.g., 1 stimulus event per second) a neuron should similarly react on each stimulus, demonstrating the simplest “stimulus-response” behavior (the same processing of the same stimuli). As the stimulus frequency increases, some stimuli can be “less” processed or missed by the neuron, and its response stability (i.e., the stability factor *S*) will decay with an increase of *f*_stim_. Indeed, such behavior has been found in about 33% of cases over all nuclei, that is, every third cell exhibits low-pass frequency dynamics (Figures 5 and 6). However, most of the neurons show different behavior.

It is known that the frequency of whisker movements plays an important role in effective perception (see, e.g., [21, 25, 26]). Previous results showed the presence of resonance properties in the firing of thalamic and cortical neurons (see review in [2]). Indeed, stimulation of a vibrissa at a given frequency can be related to its vibration during perception. Then the surface discrimination requires fine tuning of the system and a series of impulses deflecting the vibrissa can be considered a single entity. Therefore we expect an effective band-pass amplification (or filtration) of the stimuli in a given frequency band by some cells. We found that more than half (about 57%) of neurons in the trigeminal nuclei have such property. Finally, the remaining 10% of cells have no pronounced dependence on the stimulus frequency and probably these neurons perform a different task, not directly linked to the stimulus transmission. Besides, their stability factors are usually extremely low (e.g., in Figure 5, *S*_low_ ≈ 500, *S*_band_ ≈ 150, whereas *S*_nodep._ ≈ 18), which also suggests that the stimulus processing is not their primary role.

The percentage of neurons showing low-pass, band-pass, and no-dependence behaviors is quite similar across different nuclei (Figure 6(a)). This suggests that the number of neurons specializing on different tasks (e.g., border or texture detection) is also similar in Pr5, Sp5i, and Sp5o nuclei.

We also quantified the mean “optimal" stimulation frequencies of the band-pass neurons. For Pr5 and Sp5i, *F*_optm_ = 5 Hz, whereas it is about 4 Hz in Sp5o. These frequencies are close to the lower end of frequencies for whisker movements at the active exploration (4–12 Hz) [27]. Our results correlate with the studies of the amplitude of averaged neuronal responses in the somatosensory cortex where similar filtration properties have been found [21]. Thus, we can suppose that at least a part of the filtration properties observed for neurons in the somatosensory cortex can be influenced by analogous responses generated by neurons in the trigeminal complex.

## References

[1] Mehta SB, Whitmer D, Figueroa R, Williams BA, Kleinfeld D (2007). Active spatial perception in the vibrissa scanning sensorimotor system. *PLoS Biology*.

[2] Moore CI (2004). Frequency-dependent processing in the vibrissa sensory system. *Journal of Neurophysiology*.

[3] Wolfe J, Hill DN, Pahlavan S, Drew PJ, Kleinfeld D, Feldman DE (2008). Texture coding in the rat whisker system: slip-stick versus differential resonance. *PLoS Biology*.

[4] Ahissar E, Knutsen PM (2008). Object localization with whiskers. *Biological Cybernetics*.

[5] Knutsen PM, Ahissar E (2009). Orthogonal coding of object location. *Trends in Neurosciences*.

[6] Castellanos NP, Malmierca E, Nuñez A, Makarov VA (2007). Corticofugal modulation of the tactile response coherence of projecting neurons in the gracilis nucleus. *Journal of Neurophysiology*.

[7] Malmierca E, Castellanos NP, Makarov VA, Nuñez A, Porto AB, Pazos A, Buño W (2009). Corticofugal modulation of tactile responses of neurons in the spinal trigeminal nucleus: a wavelet coherence study. *Advancing Artificial Intelligence through Biological Process Applications*.

[8] Malmierca E, Castellanos NP, Nuñez-Medina A, Makarov VA, Nuñez A (2009). Neuron synchronization in the rat gracilis nucleus facilitates sensory transmission in the somatosensory pathway. *European Journal of Neuroscience*.

[9] Smith RL (1973). The ascending fiber projections from the principal sensory trigeminal nucleus in the rat. *Journal of Comparative Neurology*.

[10] Peschanski M (1984). Trigeminal afferents to the diencephalon in the rat. *Neuroscience*.

[11] Ma PM (1991). The barrelettes-architectonic vibrissal representations in the brainstem trigeminal complex of the mouse. I. Normal structural organization. *Journal of Comparative Neurology*.

[12] Friedberg MH, Lee SM, Ebner FF (2004). The contribution of the principal and spinal trigeminal nuclei to the receptive field properties of thalamic VPM neurons in the rat. *Journal of Neurocytology*.

[13] Perkel DH, Gerstein GL, Moore GP (1967). Neuronal spike trains and stochastic point processes. I. The single spike train. *Biophysical Journal*.

[14] Perkel DH, Gerstein GL, Moore GP (1967). Neuronal spike trains and stochastic point processes. II. Simultaneous spike trains. *Biophysical Journal*.

[15] Brillinger DR (1978). Developments in statistics. *Comparative Aspects of the Study of Ordinary Time Series and of Point Processes*.

[16] Jarvis MR, Mitra PP (2001). Sampling properties of the spectrum and coherency of sequences of action potentials. *Neural Computation*.

[17] Pavlov AN, Makarov VA, Mosekilde E, Sosnovtseva OV (2006). Application of wavelet-based tools to study the dynamics of biological processes. *Briefings in Bioinformatics*.

[18] Jones LM, Depireux DA, Simons DJ, Keller A (2004). Robust temporal coding in the trigeminal system. *Science*.

[19] Quian Quiroga R, Nadasdy Z, Ben-Shaul Y (2004). Unsupervised spike detection and sorting with wavelets and superparamagnetic clustering. *Neural Computation*.

[20] Pavlov A, Makarov VA, Makarova I, Panetsos F (2007). Sorting of neural spikes: when wavelet based methods outperform principal component analysis. *Natural Computing*.

[21] Garabedian CE, Jones SR, Merzenich MM, Dale A, Moore CI (2003). Band-pass response properties of rat SI neurons. *Journal of Neurophysiology*.

[22] Moreno A, Garcia-Gonzalez V, Sanchez-Jimenez A, Panetsos F (2005). Principalis, oralis and interpolaris responses to whisker movements provoked by air jets in rats. *NeuroReport*.

[23] Díaz-Quesada M, Maravall M (2008). Intrinsic mechanisms for adaptive gain rescaling in barrel cortex. *Journal of Neuroscience*.

[24] Sadeghi SG, Chacron MJ, Taylor MC, Cullen KE (2007). Neural variability, detection thresholds, and information transmission in the vestibular system. *Journal of Neuroscience*.

[25] Carvell GE, Simons DJ (1995). Task- and subject-related differences in sensorimotor behavior during active touch. *Somatosensory and Motor Research*.

[26] Harvey MA, Bermejo R, Zeigler HP (2001). Discriminative whisking in the head-fixed rat: optoelectronic monitoring during tactile detection and discrimination tasks. *Somatosensory and Motor Research*.

[27] Welker WI (1964). Analysis of sniffing of the albino rat. *Behaviour*.

